# Prevalence of Psychiatric Morbidities in Acute Coronary Heart Disease

**DOI:** 10.1155/2014/407808

**Published:** 2014-07-22

**Authors:** Saeed Shoja shafti

**Affiliations:** University of Social Welfare and Rehabilitation Sciences (USWR), Razi Psychiatric Hospital, Tehran 19646 33151, Iran

## Abstract

*Introduction.* Psychiatric problems and stresses may deteriorate the prognosis of patients with IHD. So evaluating their frequency possibly will promote our perspective regarding their vital importance in the field of consultation-liaison psychiatry. *Method and Materials.* One hundred and one (101) patients with IHD were interviewed in CCU of a general hospital by a psychiatrist to find whether there was any relationship between cardiac events and psychiatric problems or stresses. *Results.* Cardiac events were significantly more prevalent among patients with both psychiatric problems and biological risk factors (*P* < 0.05). Also, the number of patients suffering from psychiatric problems was significantly more than cases without that (*P* < 0.05). There was a significant difference between male and female patients regarding the type of stress (*P* < 0.01). 79% of total stresses were experienced by patients who had as well psychiatric problems (*P* < 0.0001). In addition, there was significantly more dysthymic disorder in the acute group of patients in comparison with major or minor depressive disorder in the chronic group (*P* < 0.001). *Conclusion.* The high prevalence of psychiatric problems and psychosocial stresses among patients with IHD deserves sufficient attention by clinicians for detection, monitoring, and management of them.

## 1. Introduction

Anxiety and depression are more prevalent in patients with cardiovascular disease (CVD) than in the general population [1]. A study of 200 patients who had suffered a first myocardial infarction (MI) found a 1-year cumulative incidence of depression of 25% [2]. Also, in patients with congestive heart failure (CHF), three studies have found the prevalence of depression to be approximately 20% [3].

Although the epidemiology of anxiety disorders in CAD is not as well studied as that of depression, the incidence of anxiety symptoms in patients with acute coronary disease in cardiac care units is approximately 50% [4]. Besides, depression is linked to a large number of major risk factors for CAD or cardiac-related mortality, including cigarette smoking, diabetes, and obesity. Prospective studies indicate that depression is an independent risk factor in the development of these behaviors or conditions and also strong independent risk factor for the development of cardiovascular disease, of acute coronary events, and of mortality from cardiac illness. In general, studies show that the relative risk of incident cardiac disease in healthy individuals with depression or symptoms of depression is about 1.5 to 2, depending on which cardiac endpoint is used [5, 6]. Also, findings of a recent study on 2,111 incident myocardial infarctions, in a large Norwegian population based cohort, showed that self-reported symptoms of depression and anxiety, especially if recurrent, were moderately associated with the risk of acute myocardial infarction [7].

In addition, three community-based studies have shown a significant relationship between anxiety disorders and sudden cardiac death. One study of 1,457 male subjects followed up over 6 years found the risk of cardiac-related death elevated almost fourfold in those with phobic anxiety [8]. Acutely stressful events, as well, dramatically increase cardiac morbidity and mortality. In a prospective study of 95,647 Finnish men and women, the risk of all-cause mortality in the first week following spousal death was two times normal. The risk of any ischemic heart disease event in that period was elevated 2.3-fold in men and 3.5-fold in women, an effect independent of age [9]. Finally, regarding relationship between type A behavior pattern (TABP)—time urgency, hostility, and achievement-striving-competitiveness—and the risk of coronary heart disease, while a 1987 meta-analysis documented the weakening of the association of type A behavior with coronary heart disease [10], the controversies still go on [11–13].

In the present study, the frequency of psychiatric problems and psychosocial stresses, which were there prior to the occurrence of acute ischemic heart events, had been estimated, to survey their likely exacerbating effect in the aforesaid morbid process.

## 2. Method and Materials

One hundred and one ischemic patients, who were admitted in the coronary care unit (CCU) of a general hospital, were elected systematically in a period of about six months (August to December 2011). After admission, diagnosis, primary medical work-up and stabilization by a cardiologist in the first few days and just before discharge from CCU, a comprehensive clinical interview was performed by a psychiatrist to determine that whether there was any psychiatric symptom or stress obtainable in the history of the cases, and If necessary, additional information was available from patient's relatives, staff, and medical practitioners, who were visiting patients on a daily basis. Psychiatric disorders had been diagnosed according to the structured clinical interview, and criteria of* Diagnostic and Statistical Manual of Mental Disorders*, 4th edition, text revision (DSM IV-TR), which was ordinary in the period of assessment. Besides, a self-made questionnaire, as a kind of assistance for examiner (psychiatrist), had been invented, which included the entire of the psychiatric symptoms plus substance abusing, characteristics of TABP, and psychosocial stresses. Priority of existence of symptoms and stresses ahead of admission was a necessity for final analysis.

*Statistical Analysis.* Data were analyzed by *Z* and chi-square (*χ* ^2^-test) formula. The statistical significance was defined as *P* value equal or less than 0.05.

## 3. Results

According to the medical data, patients were suffering from unstable angina (*n* = 41, 40.6%), acute myocardial infarction (*n* = 37, 36.6%), and congestive heart failure (*n* = 23, 22.8%).

50.5% (*n* = 51) of the patients were male and 49.5% (*n* = 50) of them were female. Acute myocardial infarction was remarkably more prevalent among men and unstable angina among women (*P* < 0.01). 77.2% (*n* = 78) of the patients were married and 22.8% (*n* = 23) were single or widowed (*P* < 0.05). Patients were between 32 and 84 years old (mean = 53/74), with 43.6% above 60. 68/3% (*n* = 69) of them had moved to capital city at some time during the last decades. 33.7% (*n* = 34) had academic educations, 7.9% (*n* = 8) were only graduated from high school, 15.8% (*n* = 16) were illiterate, and the rest had some literacy.

Most of the female patients were housekeeper (*n* = 46, 45.5%) and most of the male patients were businessman (*n* = 28, 22.8%). Also, 4% (*n* = 4) of men were jobless. 51.48% (*n* = 52) of the patients were from families with an income equal to at least 500 dollars per individual of family members per year and 48.5% (*n* = 49) of them reported less than this. 38.6% (*n* = 39) had private house and 61.4% (*n* = 67) did not have their own home and were tenant or else. There was no significant relationship between the aforesaid variables (as stresses) and cardiac events in our sample.

44.5% (*n* = 45) of the patients had both psychiatric problems and biological (somatic) risk factors (diabetes mellitus, hypertension, hyperlipidemia, or cigarette smoking) and 14.9% (*n* = 15) had neither this nor that. 21.78% (*n* = 22) had biological risk factor without any psychiatric problem and 18.81% (*n* = 19) had a psychiatric problem without any biological risk factor (Table 1). Among the aforesaid risk factors, hypertension was found in 55%, diabetes mellitus in 38%, hyperlipidemia in 37%, and cigarette smoking in 32% of the cases. 62% of the patients had more than one biologic risk factor, and this was more prevalent among female patients. Cardiac events were significantly more prevalent among patients with both psychiatric problems and biological risk factors, comparing with patients without both of them (*DF* = 1, *χ* ^2^ = 4.82, *P* < 0.05). Comorbidity of these two had increased the risk of cardiac events about threefold, and existence of psychiatric problems had increased the detrimental effects of biological risk factors about twofold, in the present assessment.

26.73% (*n* = 27) of the patients had another somatic disease in addition to their cardiac problem, with gastrointestinal diseases, especially gastroduodenitis, the most prevalent one. 55.4% (*n* = 56) had been admitted acutely and for the first time and the rest were chronic cases with previous and recurrent admissions (unstable angina and congestive heart failures).

Totally, 63.36% (*n* = 64) of cases had some kind of psychiatric problem (Table 2). Among them, 67% (*n* = 43) had some kind of depressive illness, like dysthymic disorder (*n* = 20), minor depressive disorder (*n* = 9), or major depressive disorder (*n* = 14), and 15% (*n* = 10) had an anxiety disorder, like obsessive compulsive disorder (*n* = 2), generalized anxiety disorder (*n* = 5), or phobia (*n* = 3). 17% (*n* = 11) of the patients did not have any specific syndrome and therefore were classified as not otherwise specified (NOS).

With respect to psychiatric symptoms and disregard to any specific disorder, 42.6% (*n* = 43) of the cases had depressed mood and fatigue, 48.5% (*n* = 49) had irritability and aggressiveness, 39.6% (*n* = 40) insomnia, 23.7% (*n* = 27) loss of interest and 17.9% (*n* = 18) loss of appetite, and finally 25.7% (*n* = 26) mentioned anxiety as a disturbing symptom in the last months. With respect to substances, 4% (*n* = 5) had alcohol abuse and dependency, 3% (*n* = 4) had dependency on opium, and 20.8% (*n* = 23) were dependent on cigarette smoking. So, number of patients with psychiatric problem was significantly more than cases without that (DF = 1, *χ* ^2^ = 4.82, *P* < 0.05).

Depression was about two times and anxiety 1.5 times more common in women than men (DF = 3, *χ* ^2^ = 13.35, *P* < 0.001) (Table 3).

52% (*n* = 31) of men and 47% (*n* = 28) of women had mentioned experiencing some sort of stress during the preceding days before admission (Table 4). In this regard, 58% (*n* = 18) of men and 17% (*n* = 5) of women were experiencing multiple stresses. Family stress for women and economic stress for men were the most important one (49% and 44%, resp.). This difference among men and women regarding the type of stress was statistically important (DF = 6, *χ* ^2^ = 14.06, *P* < 0.01).

Regarding relationship between psychiatric problems and stresses, we found that, in the group of patients without any psychiatric problem (*n* = 37), the ratio of patients without stress (*n* = 25) to patients with that (*n* = 12) was 2.08 : 1, and in the group with psychiatric problem (*n* = 64) the ratio for patients with stress (*n* = 41) to patients without stress (*n* = 17) was 2.76 : 1.

Therefore 79% of total stresses was experienced by patients who had psychiatric problems and this phenomenon was concomitant with fourfold increase in the risk of cardiac events, vis-à-vis patients with no comparable problem (DF = 1, *χ* ^2^ = 16.23, *P* < 0.0001) (Table 5).

Also, there was no statistical difference between male and female patients in this regard (*P* > 0.05), and with excluding psychiatric problems, there was no important difference between patients with (*n* = 59) or without (*n* = 42) stress (*P* > 0.05). But anyway in the infarction group the ratio of men with stress to comparable women was 2 : 1, while in the group of patients with unstable angina it was 1 : 1.4, and in all of the aforesaid groups the patients with stress were quantitatively more than patients without that; 57% (*n* = 19) of cases with infarction, 60% (*n* = 32) of them with unstable angina, and 56% (*n* = 8) of participants with congestive heart failure had experienced some sort of psychosocial stress. Characteristics of type A behavior as well were traceable in 32% (*n* = 11), 13% (*n* = 7), and 14% (*n* = 2) of patients with infarction, unstable angina, and congestive heart failure, respectively (Table 6). In infarction group the ratio of male to female with TABP was 2.6 : 1. But this difference was not statistically significant (DF = 2, *χ* ^2^ = 6.04, *P* < 0.25). In the infarction group of patients, among 60% (*n* = 20) of the depressive illnesses, 18% (*n* = 6) were diagnosed as major or minor depressive disorder and 42% (*n* = 14) as dysthymic disorder, and in the chronic cluster of patients (unstable angina and congestive heart failure), 51% (*n* = 17) were diagnosed as major or minor depressive disorder and 18% (*n* = 6) as dysthymic disorder, and in general 69% (*n* = 23) of the cases in the later cluster had some kind of depression (Table 7). Therefore, in this sample, the ratio of depression (major and minor) in acute infarction group to chronic ischemic ones was about 1 : 2.7, and for dysthymic disorder it was about 2.6 : 1. So, there was a significant difference (DF = 1, *χ* ^2^ = 8.26, *P* < 0.001) regarding severity of depression between acute and chronic patients. The same matter was not traceable regarding anxiety disorders.

## 4. Discussion

Cardiac events often result in disability and a change in social role function and affect the individual's perception of his or her mortality. Hence it is not surprising that depression appears to be the most common psychiatric disorder in patients with coronary artery disease [14]. Our basic goals in this research could be defined as: (1) what was the prevalence of psychiatric problems among patients suffering from acute ischemic heart disease? (2) was there any remarkable relationship between psychosocial stresses and cardiovascular events? and lastly (3) was there any perceptible detrimental influence due to TABP?

With respect to the first question and according to the findings, there was a high prevalence of psychiatric morbidities among patients with ischemic heart disease and this difference in comparison with the parallel cases without psychiatric problems was significant, and moreover such an association was generally with respect to depression. When we took into account the chronicity or acuteness of the cardiac patients, we noticed that there was a direct relationship between chronicity of cardiac disorders and increasing severity of depression or, maybe, double depression. In a relatively similar survey in India on one-hundred and thirty patients with IHD, major depressive and anxiety disorders were found in 58.4 and 36.9% patients, respectively. So around 95.4% of patients reported psychiatric symptoms, either depression, or anxiety [15], which were quantitatively far more than our findings with 42.57% for depression, 9.90% for anxiety, and 63.36% of some kind of psychiatric problem for the whole patients.

Depression and anxiety were more prevalent among female cases, in comparison with male patients (2- and 1.5-folds, resp.), and male patients without definable psychiatric problem were twofold more than female patients. These show that detrimental effects of psychiatric problems could have some gender-related characteristics and female ischemic patients possibly are more sensitive to that than male patients. Alternatively, such a difference is comparable too to the prevalence of depression and anxiety in general population.

With respect to the interplay of biological risk factors (diabetes mellitus, hypertension, hyperlipidemia, and cigarette smoking) and psychiatric problems, we found a twofold increase in pathogenicity of biological risk factors in presence of psychiatric problems and also threefold increase in cardiovascular problems when both of biological risk factors and psychiatric problems coexist. Most of the patients in this sample had both of the aforesaid problems.

Regarding stress and its interaction with cardiac events, we found that the chance of patients with psychiatric problems in comparison with cases without that, for subjective experience of stress, was 4 : 1. Therefore, 79 percent of stresses had been concentrated in cases with psychiatric problems. Consequently, we may say that psychosocial stresses and psychiatric problems had a mutual exacerbating effect on each other, or in another way we can deduce that they had absorbed each other. It is well known that mental stress-induced ischemia is more common than exercise-induced ischemia in patients with clinically stable coronary heart disease. Women, unmarried men, and individuals living alone are at higher risk for mental stress-induced ischemia [16]. Mental stress induces transient myocardial ischemia in one third to one half of patients with CAD. Ischemic responses are induced not by extremely severe emotional stress, but by behavioral challenges similar to those that might be encountered in everyday life, and they are associated with ischemia on ambulatory monitoring. Mental stress-induced ischemia is typically without pain and occurs at lower levels of oxygen demand than ischemia induced by physical exercise. It is generally not related to the severity of CAD. Stress-induced hemodynamic changes, particularly increases in systemic vascular resistance, coronary artery vasoconstriction, and microvascular changes, may all contribute to the pattern of ischemia. There is, nonetheless, considerable variability in responses to mental stress that is not understood [17]. So, this morbid process in accompany with the detrimental effects of biological risk factors can be accounted as the most harmful integration. Also, there was a significant difference between male and female patients, in the present study, with respect to the description of psychosocial stresses, who had declared family conflicts and economic problems, respectively, as their most important ones. More occurrences of cardiac events among married people, in comparison with the singles, perhaps could be attributed to the joint detrimental effect of multiple stresses. Considering more acute infarction in male patients vis-à-vis the female ones (2 : 1) and also more experiencing of stress in the infracted males (2 : 1), we may perhaps infer, once more, gender-dependent adverse effect of stress on acuteness of cardiac events, a phenomenon that was evident another time with respect to the connection between TABP and acuteness of cardiac events among male patients. On the other hand, the treatment of psychiatric disorders in patients with CVD can be challenging because of the cardiovascular side effects of many psychotropic medications as well as the potential of multiple drug-drug interactions. Moreover, many medications for CVD have psychiatric side effects.

Finally, lack of comparative non-CCU cohort, restriction of findings to a single academic center, and also small sample size were among the weak points of this assessment.

## 5. Conclusion

The high prevalence of psychiatric problems and psychosocial stresses among patients with IHD, which may act as cofactors in precipitating the pathogenicity of biological risk factors, deserves sufficient attention by clinicians for detection, monitoring, and management of them, by means of satisfactory medical and psychosocial interventions.

## Figures and Tables

**Table 1:** Prevalence of psychiatric problems and biological risk factors among patients.

Cardiac patients	Male	Female	Total
Without psychiatric problem, without biological risk factor	11	4	15
Without psychiatric problem, with biological risk factor	13	9	22
With psychiatric problem, without biological risk factor	7	12	19
With psychiatric problem, with biological risk factor	20	25	45

Total	51	50	101

**Table 2:** Prevalence of psychiatric problems among patients.

Cardiac patients	Male	Female	Total
Without psychiatric problem	27	13	37
With psychiatric problem	27	37	64

Total	51	50	101

**Table 3:** Prevalence of psychiatric problems among male and female patients.

Psychiatric problems	Male		Female		Total	
Depressive disorder	14	13.9%	29	28.7%	43	42.57%
Anxiety disorder	4	3.9%	6	5.9%	10	9.90%
Not specified	9	8.9%	2	1.9%	11	10.89%
Without psychiatric Problem	24	23.8%	13	12.9%	37	36.67%

Total	51	50.5%	50	49.5%	101	100%

**Table 4:** Prevalence of psychosocial stresses among male and female patients.

Psychosocial stresses	Male	Female	Total
Family	10	19	29
Economic	18	8	26
Vocational	9	0	9
Housing	6	2	8
Criminal	4	2	6
Health	1	2	3
Social	1	0	1

Total	49	33	82

**Table 5:** Prevalence of psychosocial stresses among patients with or without psychiatric problems.

Cardiac patients	With stress	Without stress	Total
Without psychiatric problem	12	25	37
With psychiatric problem	47	17	64

Total	59	42	101

**Table 6:** Prevalence of type A behavior among patients.

Cardiac disease	Males with type A behavior	Female with type A behavior	Male and female without type A behavior	Total
Myocardial infarction	8	3	23	34
Unstable angina	3	4	46	53
Congestive heart failure	1	1	12	14

Total	12	8	81	101

**Table 7:** Prevalence of psychiatric problems among acute and chronic ischemic patients.

Duration of cardiac disorders	Depression major + minor	Dysthymic disorder	Anxiety disorders	NOS	Total
Acute	6	14	5	6	33
Chronic	17	6	5	5	33

Total	23	20	10	11	64
